# Urinary tract diversion with gastric conduit after total pelvic exenteration for Crohn’s disease-related anorectal cancer: a case report

**DOI:** 10.1186/s40792-022-01458-x

**Published:** 2022-06-02

**Authors:** Kei Kimura, Akihiro Kanematsu, Masato Tomono, Kozo Kataoka, Naohito Beppu, Motoi Uchino, Hisashi Shinohara, Hiroki Ikeuchi, Shingo Yamamoto, Masataka Ikeda

**Affiliations:** 1 Division of Lower G.I., Department of Gastroenterological Surgery, Hyogo College of Medicine, 1-1 Mukogawa-cho, Nishinomiya, 663-8501 Japan; 2 Department of Urology, Hyogo College of Medicine, 1-1 Mukogawa-cho, Nishinomiya, 663-8501 Japan; 3 Division of Inflammatory Bowel Disease Surgery, Department of Gastroenterological Surgery, Hyogo College of Medicine, 1-1 Mukogawa-cho, Nishinomiya, 663-8501 Japan; 4 Division of Upper G.I, Department of Gastroenterological Surgery, Hyogo College of Medicine, 1-1 Mukogawa-cho, Nishinomiya, 663-8501 Japan

**Keywords:** Gastric conduit, Total pelvic exenteration, Crohn’s disease, Anorectal cancer, Laparoscopic surgery, Urinary diversion

## Abstract

**Background:**

In Japan, Crohn’s disease (CD)-related cancers occur most frequently in the anal canal. Many patients with advanced CD-related cancer require total pelvic exenteration (TPE) based on their medical history, and choosing the most effective method for urinary diversion is a major concern. We herein report the first case of CD-related cancer treatment with urinary diversion using a gastric conduit after TPE in Japan.

**Case presentation:**

A 51-year-old man with a 25 year history of CD was referred to our institution after having been diagnosed with fistulae between the rectum and urethra. Sigmoidoscopy revealed stenosis of the anal canal, and histological examination of this lesion led to a diagnosis of mucinous adenocarcinoma. Magnetic resonance imaging showed that the tumor had invaded the prostate and left internal obturator muscle, and TPE with left internal obturator muscle resection was planned. Urinary diversion was performed with a gastric conduit. The gastric conduit was created by trimming a gastric tube to a 1.5 cm width via stapled resection of the greater curvature, and the branches of the right gastroepiploic artery were preserved as feeding vessels. The ureters were raised from the mesentery on the right side of the ligament of Treitz. Ureterogastric anastomosis was performed using the Wallace technique, and the entire anastomosis was then retroperitonealized. The anastomotic site had a bleeding tendency, but hemostasis was obtained by proton pump inhibitor administration and discontinuation of enoxaparin, which had been administered to prevent venous thrombosis. No other major complications occurred, and the patient’s quality of life was recovered 6 months after surgery.

**Conclusion:**

Urinary diversion using a gastric conduit is a feasible treatment option for patients with CD-related anorectal cancer requiring TPE.

## Background

Crohn’s disease (CD)-related colorectal cancers occur at anorectal sites more frequently in Japan than in other countries. CD-related anorectal cancers include many advanced cases with T4 invasion, have aggressive histological features such as mucinous and poorly differentiated types, and are known to have a poor prognosis [1, 2]. The mainstay of treatment for CD-related anorectal cancer is surgical intervention because of the insufficient outcomes of preoperative treatments such as chemotherapy and chemoradiotherapy [3].

However, surgical treatment may be problematic because many anorectal cancers exhibit diffuse infiltration or extraluminal progression within a narrow pelvis and concomitant anorectal fistulae. These patients require total pelvic exenteration (TPE) with extensive perineal skin excision for curative resection [4]. Generally, urinary diversion is performed with an ileal conduit, but the ileum cannot be used in patients with CD-related anorectal cancer because it is the site of predilection for CD [5]. Therefore, cutaneous ureterostomy or nephrostomy is chosen for urinary diversion.

Self-care of a cutaneous ureterostomy is difficult because urine is drained from two stomas, and one of the major problems is that the urinary stent cannot be removed because of the ureteral stricture. Some problems associated with a nephrostomy include the location of catheters in the back, the need for frequent replacement, and the risk of urinary infection, which significantly deteriorates patients’ quality of life (QOL) compared with an ileal conduit [6]. CD can affect the entire gastrointestinal tract from the mouth to the anus. In the duodenum and ileum, strictures are found in many cases and may prevent passage of the endoscope; however, stenosis rarely occurs in the stomach [7].

We herein describe the first case of urinary diversion in Japan using a gastric conduit after TPE for CD-related anorectal cancer.

## Case presentation

**Fig. 1:**
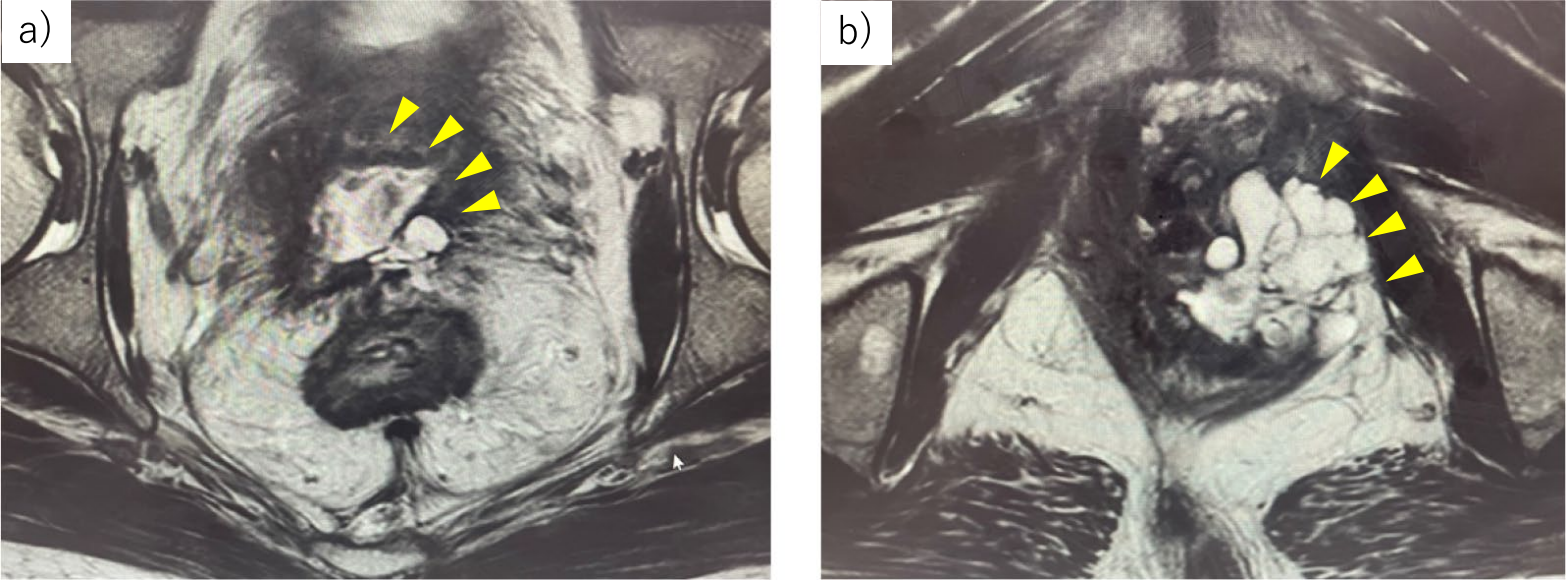
T2-weighted axial magnetic resonance images of the pelvis **a** The tumor, including a mucin pool, had spread to the prostate (yellow arrowheads). **b** Beyond the levator ani muscle, the tumor had invaded the obturator internus muscle on the left side (yellow arrowheads)

A 51-year-old man with a 25-year history of CD was referred to our institution after having been diagnosed with fistulae between the rectum and urethra. Sigmoidoscopy revealed stenosis of the anal canal with mucin production, and histological examination of this lesion led to a diagnosis of mucinous adenocarcinoma. Computed tomography showed no lymph node or distant metastasis. Magnetic resonance imaging confirmed that the tumor involved the prostate and urethra and that fistulae had penetrated through the levator ani muscle and invaded the left obturator internus muscle (Fig. 1a, b). After a discussion at a multidisciplinary team meeting, we decided to perform laparoscopic TPE-combined resection of the left obturator internus muscle and a wide perineal skin incision concomitant with transanal total mesorectal excision. After removal of the tumor, reconstruction of the soft tissue and skin defect was planned using a vertical rectus abdominis myocutaneous flap. Additionally, urinary diversion was planned using a gastric conduit instead of an ileal conduit. This surgical intervention was approved by the Institutional Review Board of the Hyogo College of Medicine (ID: 80). Informed consent was obtained from the patient.

Five ports were placed as follows: 12 mm ports at the umbilicus, upper left quadrant, and lower right quadrant and 5 mm ports at the upper right quadrant and lower left quadrant. The abdominal phase of the operation was conducted using a medial-to-lateral approach. Next, we started the pelvic phase with dissection of the posterior wall of the rectum, which proceeded with the total mesorectal excision line until the levator ani muscle was visible.

Lateral lymph node dissection was performed to obtain a clear lateral margin. The external iliac vessels, psoas muscle, obturator internus muscle, and pubic bone were exposed, and the parietal fascia was identified. The ureterohypogastric fascia and vesicohypogastric fascia were then mobilized, and the umbilical artery and inferior vesical vessels were ligated at the root of the internal iliac vessels. The dissection was continued distally toward the internal pudendal vessels. The pudendal vessels and the inferior gluteal vessels were then ligated at the infrapiriform foramen.

**Fig. 2:**
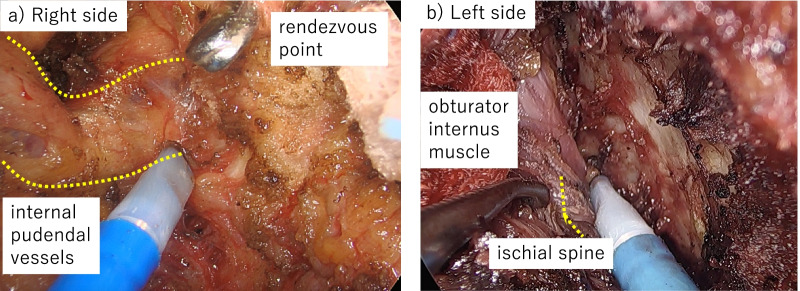
Transanal total mesorectal excision **a** The rendezvous point is used to identify the internal pudendal vessels (yellow dashed line) from the perineal side. **b** The ischial spine (yellow dashed line) was identified, and the obturator internus muscle was dissected, exposing the bone

**Fig. 3:**
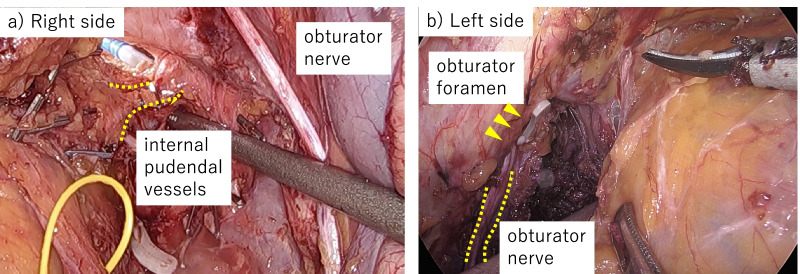
Laparoscopic view **a** The rendezvous was performed with the perineal side based on the internal pudendal vessels (yellow dashed line). **b** The left internus obturator muscle was incised using the obturator nerve (yellow dashed line) and foramen (yellow arrowheads) as guides

At the same time, the transperineal approach was taken. The perineal skin including the anorectal fistulae was incised, the ischiorectal fat was dissected, and the rectal lumen was tightly closed and washed. A multi-access port (GelPOINT® Mini; Applied Medical, Rancho Santa Margarita, CA, USA) was then placed. First, the dissection proceeded posteriorly toward the right laterally. Using the coccyx and gluteus maximus muscle as landmarks, the levator ani muscle was dissected and the retrorectal space was entered. Next, right-side dissection was performed along the levator ani muscle, and we entered the right obturator space and identified the internal pudendal vessels, where the endopelvic fascia was incised to proceed to the pelvic cavity (Fig. 2a). Finally, left sidewall dissection along with resection of the left-side obturator internus muscle was performed with the sciatic notch as the guide (Fig. 2b). From the abdominal side, in cooperation with the perineal team, we recognized the obturator foramen, the most important landmark that goes deeper to resect the obturator muscle than with the conventional TPE procedure (Fig. 3a, b). We then proceeded and completed the dissection.

After both ureters were transected, the anterior dissection was performed, and the dorsal vein complex and urethra were ligated using a linear stapler. A 10-cm incision was made and the specimen was extracted. Finally, the plastic surgeons performed the perineal reconstruction using the rectus abdominis muscle. At the same time, the urologist performed a urinary diversion through the abdominal wound.

### Urinary diversion

**Fig. 4:**
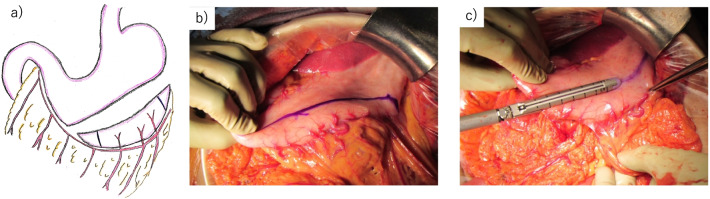
Trimming for establishment of gastric conduit **a** The incision line for the gastric conduit was determined. **b** The gastric conduit was marked to establish a tube approximately 1.5 cm wide. **c** The stomach was cut away using a linear stapler to keep it straight toward the fornix

**Fig. 5:**
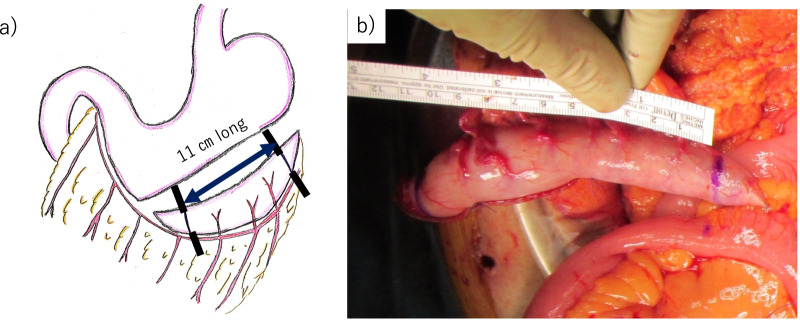
Creation of gastric conduit **a** Both ends were trimmed, and the gastric conduit was 11 cm long. **b** The gastric conduit was 11 cm in diameter

**Fig. 6:**
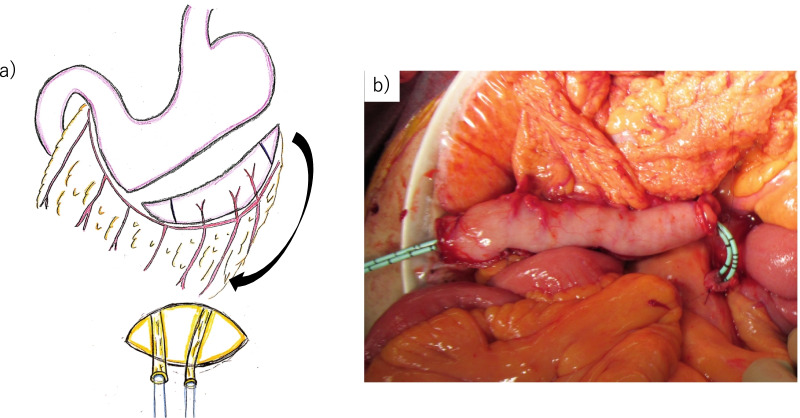
Ureterogastric anastomosis **a** Both ureters were pulled out from the retroperitoneum to the abdominal side through the mesentery of the small intestine on the right side of the ligament of Treitz. **b** Ureterogastric anastomosis using the Wallace technique was performed with isoperistaltic anastomosis. The anastomotic site was retroperitonealized

1. Both ureters were pulled out from the retroperitoneum to the abdominal side through the mesentery of the small intestine on the right side of the ligament of Treitz.
2. The branch of the right gastroepiploic artery was preserved as a feeding vessel. From 2 cm above the pylorus, the stomach was transected vertically using a linear stapler, and a 1.5-cm-wide conduit along the greater curvature was created toward the fornix (Fig. 4a–c). The gastric conduit was 11 cm long (Fig. 5a, b). The resection stump was buried.
3. Ureterogastric anastomosis using the Wallace technique was performed with isoperistaltic anastomosis (Fig. 6a, b).
4. The entire ureterogastric anastomosis was placed retroperitoneally and covered by the greater omentum and retroperitoneum. The distal end of the conduit was brought up through the right upper quadrant stoma site, and the stoma was everted.

The operating time was 883 min, and the blood loss volume was 1520 mL. Pathologic examination of the specimen showed a negative circumferential resection margin. Postoperatively, the patient developed Clavien–Dindo grade II functional dyspepsia and grade II ureterogastric anastomotic bleeding, which required administration of a proton pump inhibitor and discontinuation of enoxaparin. Grade III lymphatic leakage requiring computed tomography-guided puncture was also observed. The patient was discharged from the hospital 51 days after surgery. Six months after the surgical resection, he showed no evidence of recurrence.

We assessed the patient’s perioperative QOL using the Functional Assessment of Cancer Therapy-Colorectal (FACT-C) instrument [8]. His QOL was lower at 1 month after the surgery and recovered at 6 months after the surgery.

## Discussion

We have herein reported a case of urinary diversion using a gastric conduit after TPE for CD-related anorectal cancer. This intervention is the first to be reported in Japan to date, and it showed good results in terms of safety and efficacy.

CD-related cancers in Japan are characterized by a high incidence in the anorectal canal. The main problem in the treatment of CD-related anorectal cancer is that the ileum, which is the site of predilection for CD, cannot be used as the urinary diversion after TPE [9, 10].

No reports have described the problems posed by urinary diversion after TPE in patients with CD-related anorectal cancer. However, our institution experienced seven cases of extended surgery that required excision of adjacent organs for treatment of CD-related anorectal cancers from 2017 to 2021 (Table 1), and the method of urinary diversion after TPE was a very important issue. Five patients underwent TPE, and until the present case, a cutaneous ureterostomy was performed in one patient and a nephrostomy was performed in four patients. The patient with the cutaneous ureterostomy could not undergo stent removal because of ureteral stenosis. In two of the patients with a nephrostomy, the procedure could not be performed intraoperatively because they did not have hydronephrosis, and repuncture was required the day after the surgery. Puncture was also very difficult in the other two patients. In addition, these patients were in the working age population and experienced a markedly deteriorated QOL because cutaneous ureterostomy and nephrostomy are known to be more difficult to manage than an ileal conduit.

**Table 1:** Crohn’s disease-related anorectal cancer requiring combined resection of adjacent organs in our institution

Sex	Age (years)	Type of operation	Combined resection	Urinary diversion	Operative time (min)	Blood loss (mL)	Complication (C–D grade)	Postoperative length of stay (days)
Female	38	TPE	Vagina	Cutaneous ureterostomy	798	1750	II	41
Male	40	TPES	Sacrum (below S4) Left obturator internus muscle	Nephrostomy	998	735	IIIa (ileus)	42
Male	36	TPE	Penis Right obturator internus muscle	Nephrostomy	979	230	II	52
Male	47	TPES	Sacrum (below S4)	Nephrostomy	936	320	IIIa (lymphatic leakage)	40
Male	48	TPE	Left obturator internus muscle	Nephrostomy	783	530	IIIa (lymphatic leakage)	42
Male	60	APR	Prostate Seminal vesicle	Cystostomy	886	840	II	32
Male	50	TPE	Left obturator internus muscle	Gastric conduit	883	1520	IIIa (lymphatic leakage)	51

*C–D* Clavien–Dindo, *TPE* total pelvic exenteration, *TPES* total pelvic exenteration with sacrectomy, *APR* abdominal perineal resection

The feasibility of using a gastric conduit was reported in 1978 [11]. However, reports on gastric conduits have since been scarce except for a recent one indicating the usefulness of a gastric conduit in patients with inflammatory bowel disease [12]. We therefore performed urinary diversion using a gastric conduit in our patient. Upper gastrointestinal and urologic surgeons worked together to perform this surgical intervention, and no problems arose during the procedure. With respect to complications, the patient developed grade II functional dyspepsia and was prescribed rikkunshito, a traditional Japanese medicine. Additionally, by receiving supportive therapy including nutritional guidance regarding food intake, the patient’s symptom resolved before he was discharged. Grade II anastomotic bleeding also occurred. Endoscopy from the gastric conduit showed ureterogastric anastomosis, bleeding of which is difficult to control by endoscopy; however, the patient was treated with a proton pump inhibitor and discontinued enoxaparin to prevent postoperative venous thrombosis. The patient’s QOL improved at 6 months after surgery. No other severe postoperative complications occurred.

## Conclusion

When an ileal conduit cannot be used for urinary diversion in patients with CD-related anorectal cancer, urinary diversion using a gastric conduit can be a feasible and valuable treatment option.

## Data Availability

Not applicable.

## Abbreviations

CD: Crohn’s disease
TPE: Total pelvic exenteration
QOL: Quality of life
